# Duodenal perforation after retroperitoneal laparoscopic radical nephrectomy for ruptured malignant renal PEComa with spontaneous retroperitoneal hemorrhage: a case report

**DOI:** 10.3389/fonc.2026.1792626

**Published:** 2026-04-02

**Authors:** Juncheng Guo, Xun Cai, Shiju Shen, Xianzhou Jiang, Zhaocun Zhang, Benkang Shi

**Affiliations:** 1 Department of Urology, Qilu Hospital of Shandong University, Jinan, China; 2 Department of Urology, The People’s Hospital of Huantai, Zibo, China; 3 Institute of Physiology and Science-Information Technology (IT), Charité – Universitätsmedizin Berlin, Berlin, Germany

**Keywords:** duodenal injury, laparoscopic radical nephrectomy, PEComas, urine amylase, Wunderlich syndrome

## Abstract

**Background:**

Perirenal perivascular epithelioid cell tumor (PEComa) is a rare mesenchymal neoplasm that can cause spontaneous retroperitoneal hemorrhage. Duodenal perforation after nephrectomy is rare but potentially life-threatening.

**Case presentation:**

We report a 52-year-old man with a right renal mass and spontaneous retroperitoneal hematoma who underwent retroperitoneal laparoscopic radical nephrectomy. Intraoperatively, a large, organized hematoma and dense adhesions were found between the renal upper pole and the second portion of the duodenum. Shortly after surgery, he developed acute abdominal pain with peritoneal signs, marked leukocytosis, rapidly elevated procalcitonin, and markedly elevated urine amylase (hyperamylasuria). Emergency laparotomy revealed a small duodenal perforation, which was treated with debridement, primary repair with omental reinforcement, drainage, and jejunal feeding tube placement. He recovered gradually​ and was discharged on postoperative day 17. Pathology confirmed malignant perirenal PEComa with extensive intratumoral hemorrhage, capsular rupture, and a high Ki-67 index; follow-up CT at 2 months showed no residual fluid collection.

**Conclusions:**

This case indicates malignant perirenal PEComa as a rare cause of Wunderlich syndrome and underscores the need to anticipate duodenal injury in right upper-pole renal tumors with massive hematoma or severe adhesions, consider early conversion for safer duodenal mobilization, and promptly evaluate persistent postoperative inflammation with markedly elevated urine amylase (hyperamylasuria).

## 1 Introduction

Perivascular epithelioid cell tumors (PEComas) are a rare group of mesenchymal neoplasms composed of distinctive perivascular epithelioid cells that coexpress melanocytic and smooth muscle markers (1, 2). They have been described in a wide range of anatomical sites, including the kidney, liver, uterus, gastrointestinal tract, lung and soft tissue (3–6). Although most PEComas are considered benign or of uncertain malignant potential, a subset behaves aggressively with local recurrence and distant metastasis, making early recognition and appropriate management clinically important (7).

Renal PEComas are uncommon and often present with nonspecific clinical and radiologic features. Patients may be asymptomatic or exhibit flank pain, hematuria, or a palpable mass, and some cases initially manifest as spontaneous retroperitoneal hemorrhage or Wunderlich syndrome due to tumor rupture (8–10). On imaging, renal PEComas frequently mimic other renal neoplasms such as angiomyolipoma or renal cell carcinoma, particularly when associated with hemorrhage, fat-poor components or atypical enhancement patterns (11, 12). Consequently, a definitive diagnosis typically relies on histopathological evaluation combined with an appropriate immunohistochemical profile, including expression of melanocytic markers such as HMB-45 and Melan-A together with smooth muscle markers (11).

Duodenal injury following retroperitoneal laparoscopic radical nephrectomy is an exceptionally rare but potentially life-threatening complication, particularly in the setting of a ruptured renal tumor with dense adhesions to adjacent structures (12). Here, we report a case of malignant PEComa of the right kidney complicated by duodenal perforation after retroperitoneal laparoscopic radical nephrectomy. This case highlights the diagnostic challenges of malignant renal PEComa and underscores the need for vigilance regarding occult duodenal injury in patients with tumor rupture and extensive perirenal inflammation.

Here, we describe a case of malignant renal PEComa presenting as spontaneous retroperitoneal hemorrhage and Wunderlich syndrome, complicated by delayed duodenal perforation after retroperitoneal laparoscopic radical nephrectomy. This report highlights the diagnostic challenges posed by this rare clinical scenario and underscores the importance of recognizing persistent systemic inflammation and markedly elevated urine amylase as potential early indicators of retroperitoneal gastrointestinal leakage, prompting timely imaging and re-exploration.

## 2 Case presentation

### 2.1 Clinical presentation and initial evaluation

A 52-year-old man with no significant past medical or family history was admitted to the Department of Urology at our hospital on 1 March 2025 for evaluation of a right renal mass and spontaneous retroperitoneal hemorrhage detected on imaging. He reported a 2-week history of right flank pain but denied gross hematuria, fever or weight loss. On admission, he was hemodynamically stable, with a blood pressure of 117/79 mmHg and a heart rate of 78 beats/min. Physical examination revealed right flank tenderness without peritoneal signs or a palpable abdominal mass.

Baseline laboratory tests showed borderline anemia and activation of the coagulation–fibrinolytic system. Hemoglobin was 13.3 g/dL (red blood cell count 4.16×10^12/L, hematocrit 39.0%), fibrinogen was 4.33 g/L and D-dimer was 3.40 μg/mL. These findings were considered to reflect activation of coagulation and fibrinolysis in the setting of a ruptured renal tumor with retroperitoneal hematoma rather than systemic coagulopathy. Other routine hematologic and biochemical parameters were within normal limits or only mildly altered.

**Figure 1:**
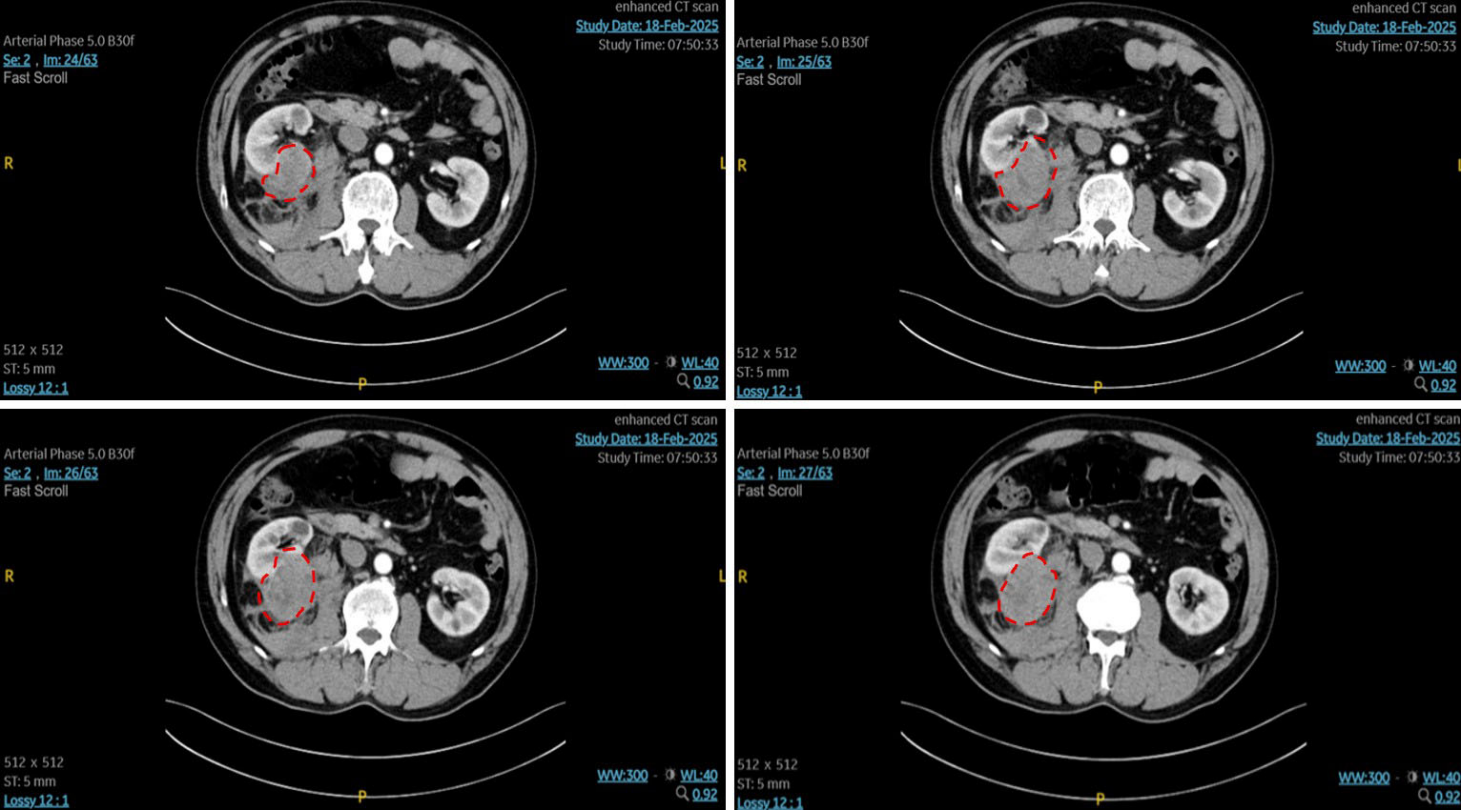
Contrast-enhanced CT (axial view). An ill-defined, heterogeneous mass is seen in the right kidney, measuring approximately 64 × 50 mm, with heterogeneous enhancement on contrast-enhanced imaging. Extensive surrounding patchy and band-like areas of slightly increased attenuation are present, consistent with peritumoral hemorrhage. The perirenal fascia is diffusely thickened, and the interfaces with the duodenum and psoas major muscle are indistinct. Overall, these findings suggest rupture of a right renal tumor with hemorrhage.

**Figure 2:**
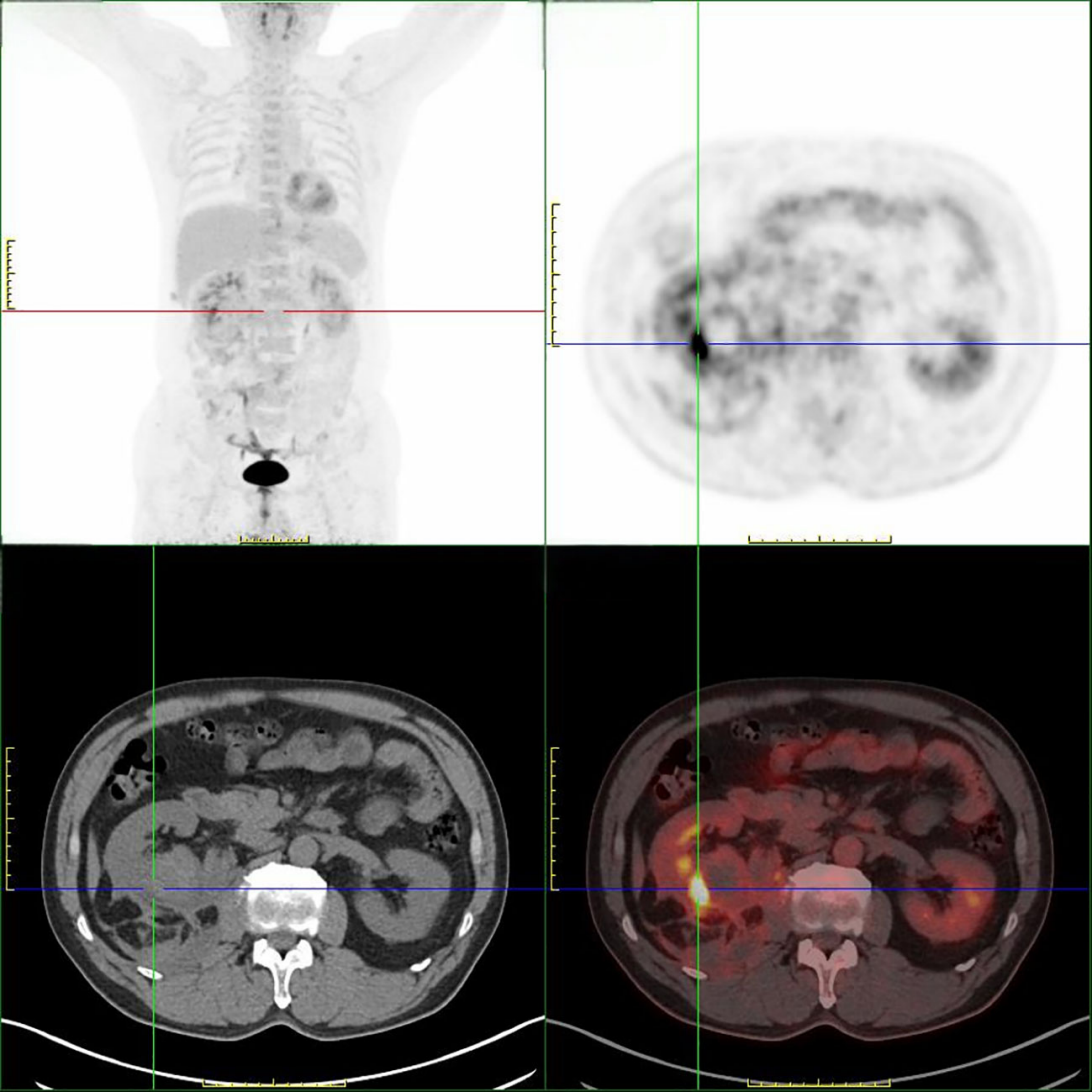
[18F]FDG PET/CT findings. Whole-body maximum-intensity-projection (MIP) image and regional axial CT, PET, and fused PET/CT images of the abdomen. The fused abdominal images show a focal area of increased [18F]FDG uptake posterior to the lower pole of the right kidney with an indistinct interface with adjacent structures, consistent with a neoplastic lesion with imaging features suggestive of rupture.

Contrast-enhanced computed tomography (CT) of the abdomen demonstrated a right renal mass measuring approximately 64 x 50 mm, predominantly located in the posterior lower pole, with heterogeneous enhancement and a large perirenal and retroperitoneal hematoma extending cranially to the upper pole, where subsequent intraoperative findings confirmed capsular rupture and dense adhesions to the duodenum (**Figure 1**). Additionally, [18F]FDG PET/CT was performed, which revealed a focal area of increased uptake posterior to the lower pole of the right kidney (**Figure 2**). The findings were compatible with spontaneous tumor rupture. There was no obvious intraperitoneal free air and no evidence of distant metastasis. As the patient remained clinically stable under close observation, elective retroperitoneal laparoscopic radical nephrectomy was planned.

### 2.2 Operative findings and procedure

On 4 March 2025, the patient underwent right retroperitoneal laparoscopic radical nephrectomy. Intraoperatively, a large organized hematoma was found around the right kidney, with capsular rupture of the tumor at the upper pole and dense adhesions between the hematoma, the upper pole of the kidney and the second portion of the duodenum. The kidney and tumor were carefully dissected from the surrounding tissues and removed en bloc. Meticulous hemostasis was achieved. No obvious bowel perforation or serosal defect was identified despite inspection of the adjacent duodenum. A retroperitoneal drain was placed near the nephrectomy bed.

### 2.3 Postoperative course and diagnostic assessment

In the immediate postoperative period, the patient initially remained hemodynamically stable and reported no specific complaints. However, on the afternoon of postoperative day (POD) 1 (5 March 2025), he developed sudden severe abdominal pain with board-like abdominal rigidity and fever. Serial laboratory tests demonstrated a marked inflammatory response. On 5 March, the white blood cell (WBC) count was 20.28×10^9/L with 92.6% neutrophils, and serum procalcitonin (PCT) was 2.020 ng/mL. Despite broad-spectrum antibiotics and supportive care, inflammatory markers remained significantly elevated over the following days, with WBC levels still raised (15.35×10^9/L with 87.8% neutrophils on 8 March) and PCT increasing further to 10.66 ng/mL on 7 March. Liver function tests were mildly abnormal, with transient elevations of alanine aminotransferase, γ-glutamyltransferase and alkaline phosphatase. The trends in key laboratory parameters, including leukocytosis, procalcitonin levels and urine amylase, are summarized in **Table 1**.

**Table 1:** Trends in key laboratory parameters during hospitalization.

Date	POD (day)	WBC (×10^9^/L)	PCT (ng/mL)	AMYL (IU/L)	Main clinical events
(March 2025)					
1	–	13	–	–	Admission; spontaneous retroperitoneal hemorrhage detected on CT
4	0	–	–	–	Right retroperitoneal laparoscopic radical nephrectomy
5	1	20.28(92.60% neutrophils)	2.02	18,700	Acute abdomen with SIRS; emergency duodenal repair
7	3		10.66	–	Persistent systemic inflammatory response; peak PCT level
8	4	15.35(87.80% neutrophils)	5.39		Persistent SIRS after duodenal repair, PCT decreasing

POD, postoperative day; WBC, white blood cell count; PCT, procalcitonin; AMYL, urine amylase; SIRS, systemic inflammatory response syndrome.

### 2.4 Reoperation and short-term outcome

In view of the persistent systemic inflammatory response together with unexplained hyperamylasuria and worsening abdominal signs, the patient was taken back to the operating room for exploratory laparotomy. Intraoperatively, a small perforation was identified in the second portion of the duodenum, close to the area where the ruptured renal tumor and organized hematoma had been adherent preoperatively. The perforation margins were debrided, and the defect was closed with interrupted absorbable sutures, reinforced with an omental patch. Wide retroperitoneal and intraperitoneal drainage was established, and a feeding jejunostomy tube was placed for postoperative enteral nutrition.

**Figure 3:**
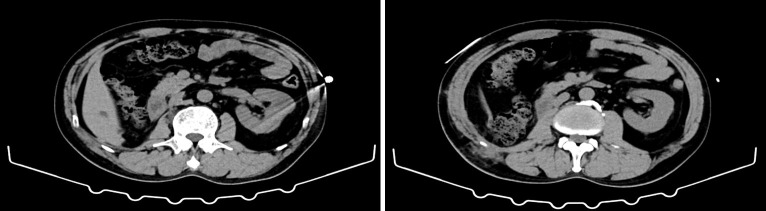
Non-contrast follow-up CT (axial view) 2 months after surgery: The right kidney is absent postoperatively, and postoperative scar changes are noted in the right lumbar region. The anatomy at the operative site is mildly distorted, with strand-like linear densities. Several small lymph nodes are seen in the abdominal cavity and retroperitoneum. No definite intra-abdominal or retroperitoneal fluid collection is identified.

Postoperatively, the patient was managed with bowel rest, continuous gastrointestinal decompression, broad-spectrum antibiotics, proton pump inhibitors and jejunostomy feeding. His clinical condition gradually improved, with progressive resolution of fever and abdominal symptoms. Inflammatory markers and PCT levels decreased steadily; by 15 March 2025, serum PCT had fallen to 0.225 ng/mL. Follow-up imaging confirmed resolution of the retroperitoneal collection and complete healing of the duodenal perforation, and the patient was able to resume oral intake (**Figure 3**).

### 2.5 Histopathological findings and follow-up

**Figure 4:**
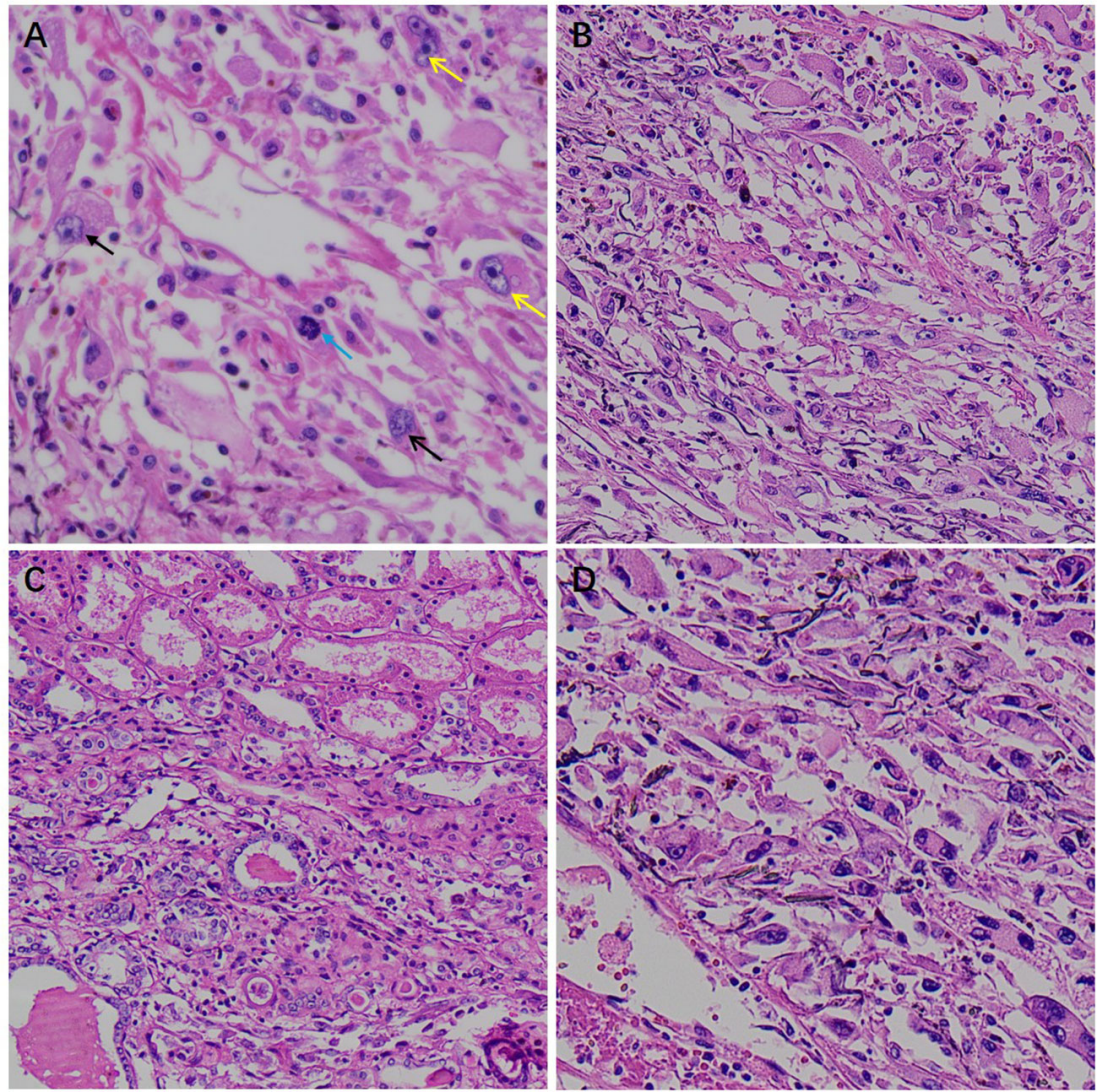
Histopathological features of malignant renal PEComa on hematoxylin–eosin (H&E) staining. **(A)** High-power view showing marked cytologic atypia: black arrows indicate enlarged pleomorphic nuclei; yellow arrows indicate prominent nucleoli; blue arrows indicate mitotic figures. **(B)** Tumor cells display a nested and sheet-like growth pattern with relatively compact arrangement. **(C)** Representative areas showing infiltrative tumor growth into the surrounding tissue. **(D)** Pronounced tumor cell heterogeneity is observed, including binucleated/multinucleated tumor cells and striking nuclear pleomorphism (variable nuclear size and irregular nuclear contours, with occasional bizarre/giant nuclei), irregular nuclear membranes, and coarse, hyperchromatic chromatin, supporting significant cellular atypia and intratumoral heterogeneity.

**Figure 5:**
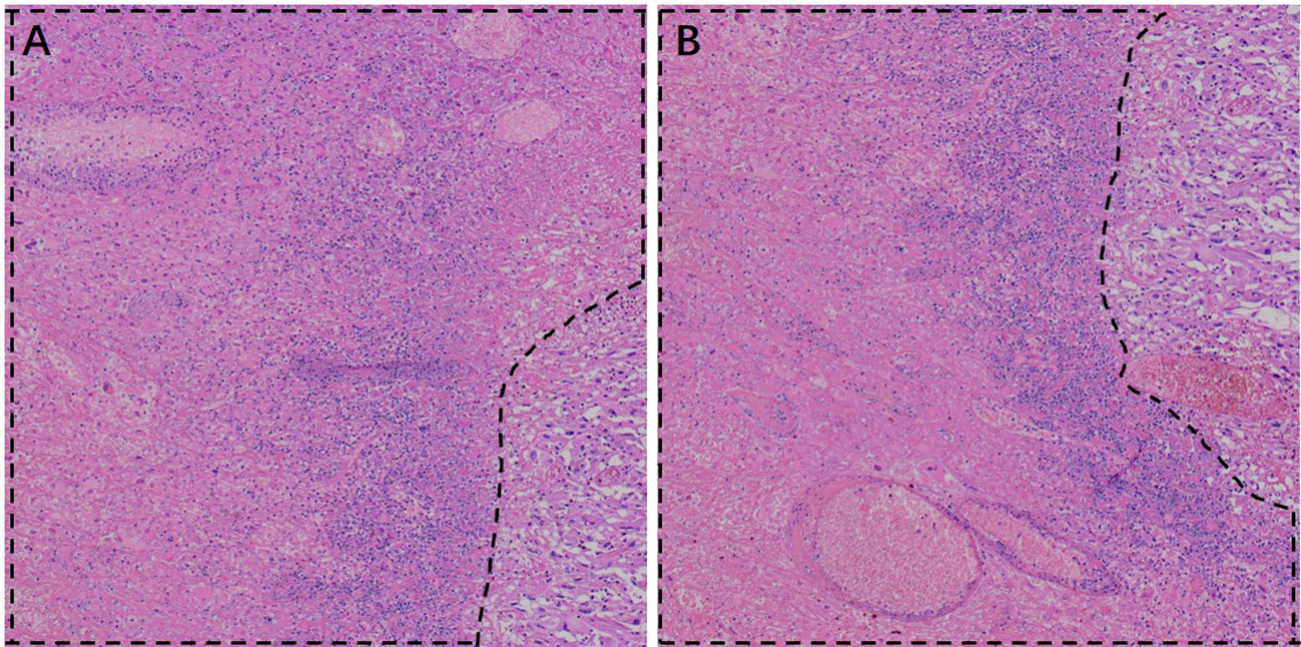
Histopathological features of malignant renal PEComa on hematoxylin–eosin (H&E) staining. **(A, B)** The dashed outlines indicate areas of tumor necrosis.

In this case, the cut surface of the right renal tumor measured approximately 5.5 × 5.0 cm. The lesion was poorly circumscribed, showed an infiltrative growth pattern, and extended into the perirenal adipose capsule, without involvement of the renal pelvis or renal sinus. On hematoxylin–eosin staining, the tumor was composed predominantly of perivascular epithelioid cells, partly arranged in nests and sheets, and radially distributed around thin-walled vessels, with marked cytologic atypia, necrosis, and infiltrative growth (**Figures 4**, **5**). The tumor cells were relatively large with abundant cytoplasm that was clear or eosinophilic, and exhibited marked cytologic atypia. The nuclei were enlarged and hyperchromatic with irregular contours; bizarre nuclei were present, with coarse granular chromatin and prominent nucleoli. Foci of coagulative necrosis and atypical mitotic figures were identified, indicating high proliferative activity. Although no definite lymphovascular or perineural invasion was observed and the ureteral and vascular margins were negative, the infiltrative growth, pronounced atypia, necrosis, and mitotic activity were morphologically consistent with malignant PEComa.

**Figure 6:**
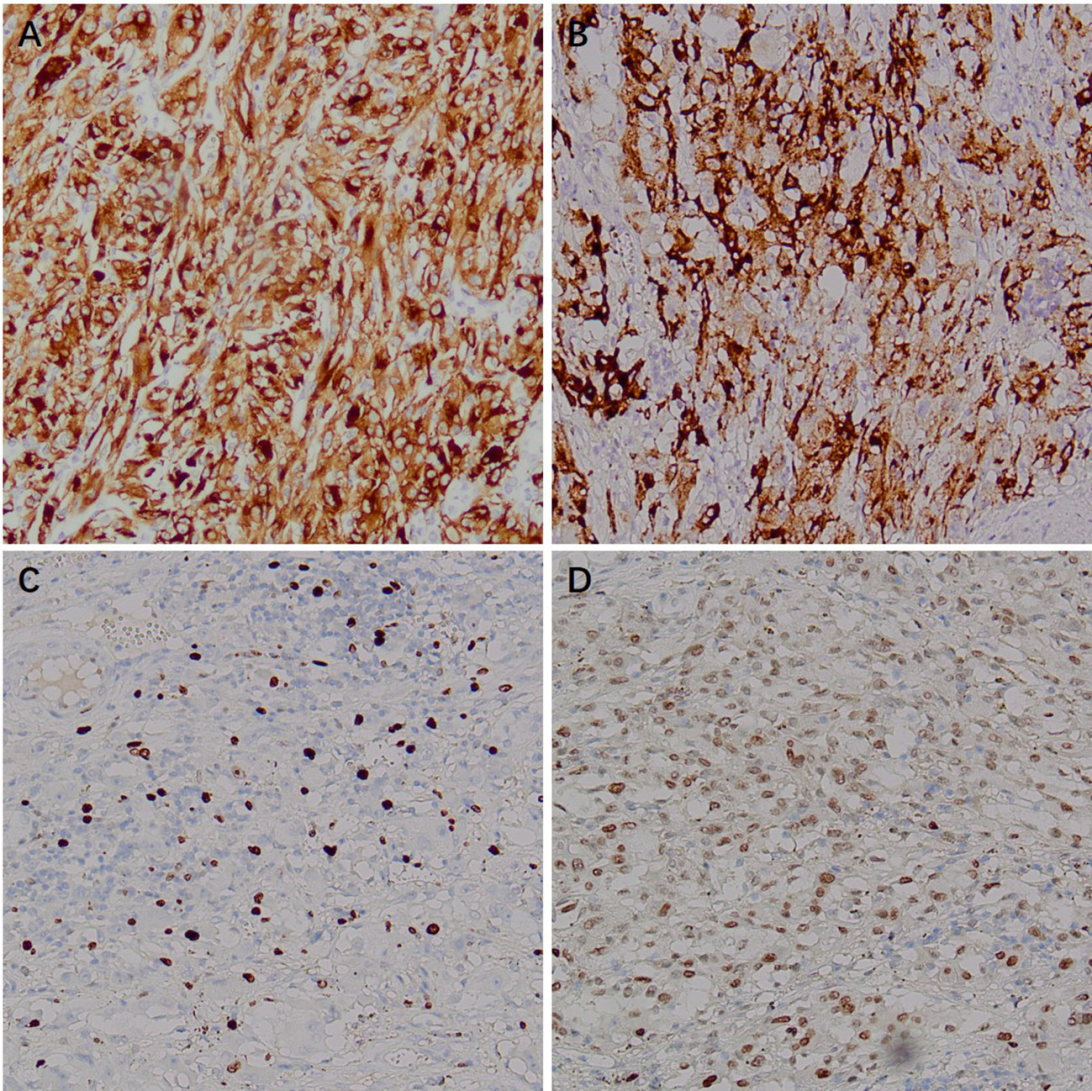
Immunohistochemical (IHC) staining of malignant renal PEComa. **(A)** Melan-A: tumor cells show positive staining. **(B)** HMB-45: tumor cells show positive staining. **(C)** Ki-67: nuclear positivity in tumor cells, with a proliferative index of approximately 20%. **(D)** TFE3: nuclear positivity in tumor cells.

**Figure 7:**
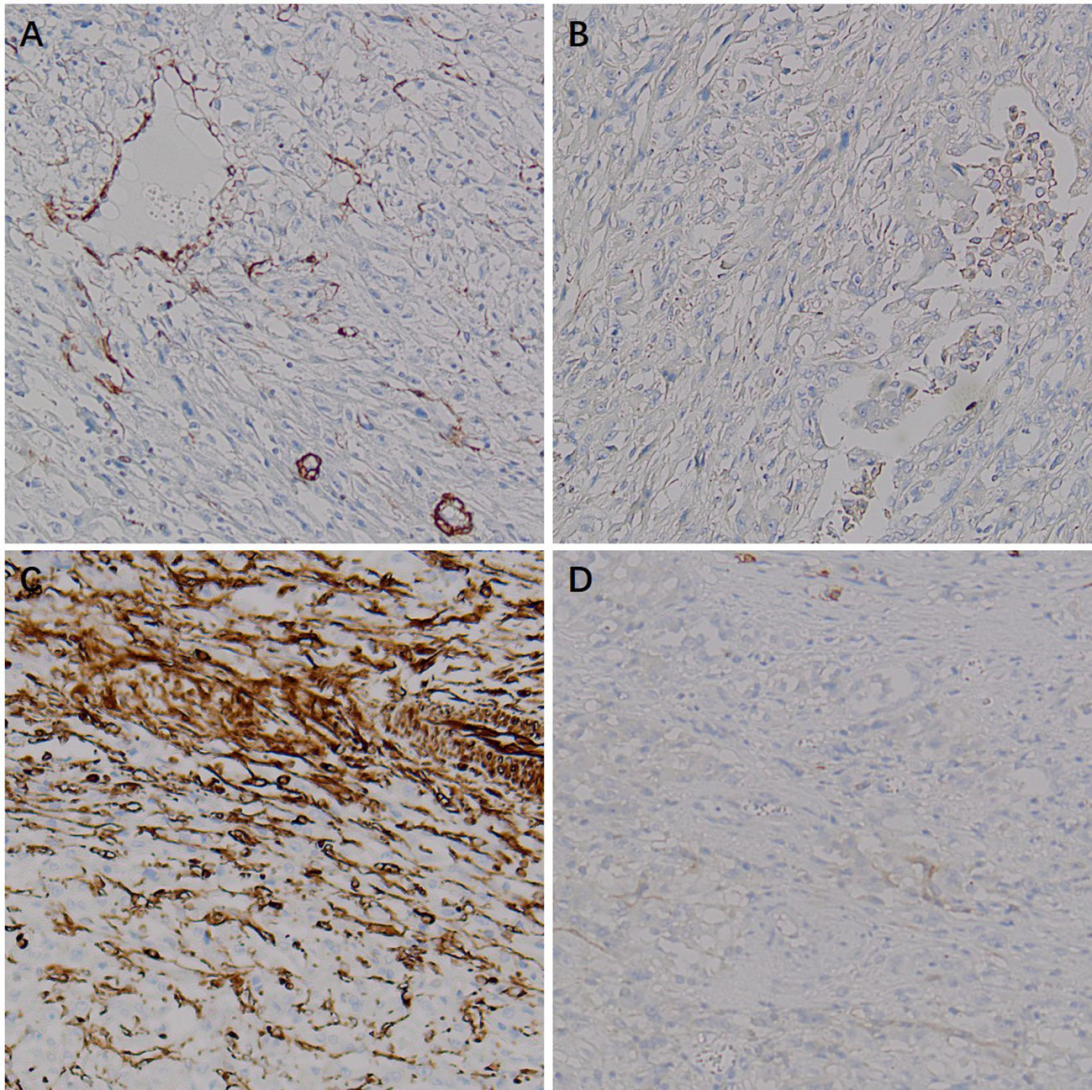
Immunohistochemical (IHC) staining of malignant renal PEComa (smooth muscle/mesenchymal markers). **(A)** SMA: weak positive staining in tumor cells. **(B)** Desmin: weak positive staining in tumor cells. **(C)** Vimentin: positive staining in tumor cells. **(D)** CD117: negative staining in tumor cells.

**Figure 8:**
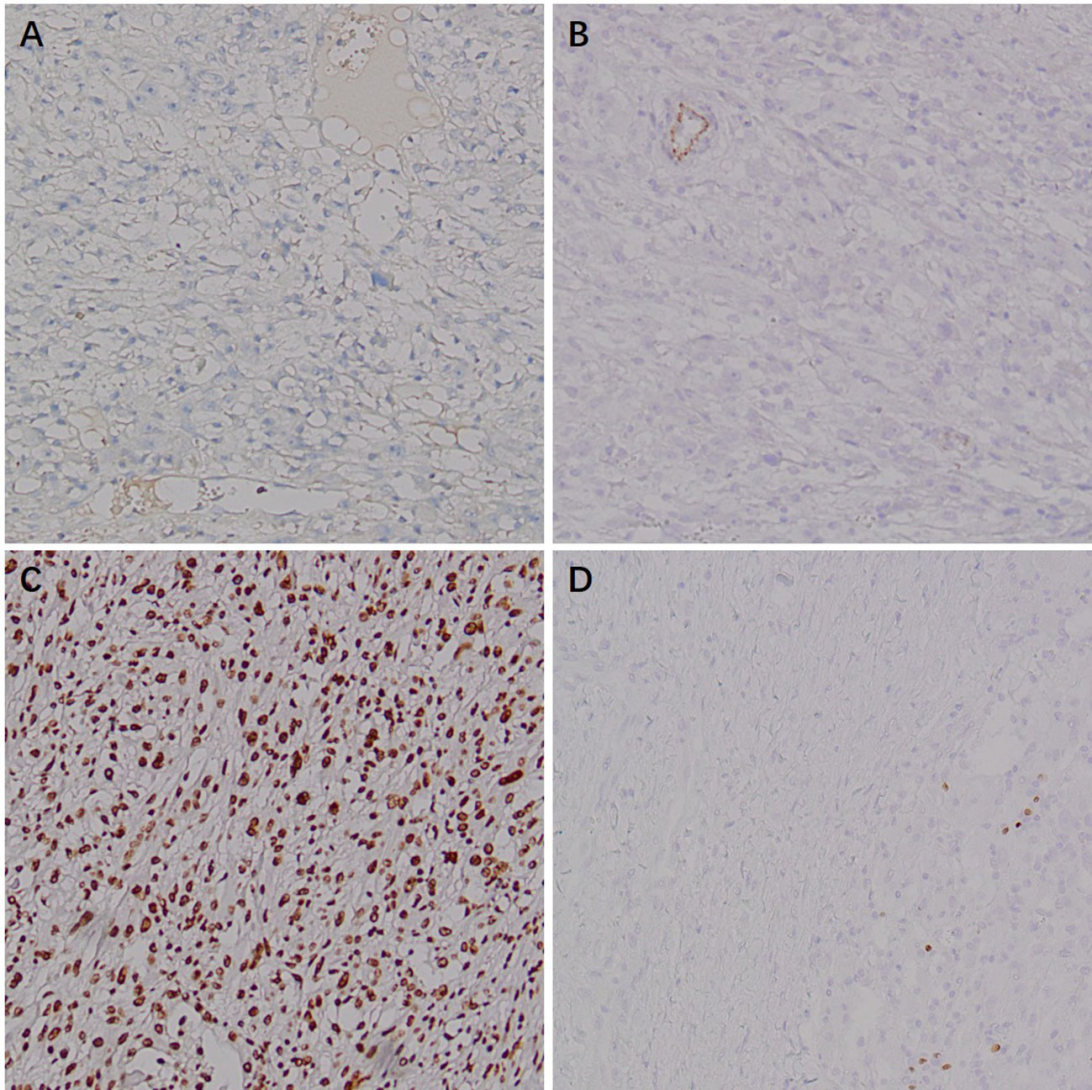
Immunohistochemical (IHC) staining of malignant renal PEComa (markers for differential diagnosis of renal tumors). **(A)** RCC: negative staining in tumor cells. **(B)** CAIX: negative staining in tumor cells. **(C)** INI-1: retained nuclear expression (positive) in tumor cells. **(D)** PAX8: negative staining in tumor cells.

**Figure 9:**
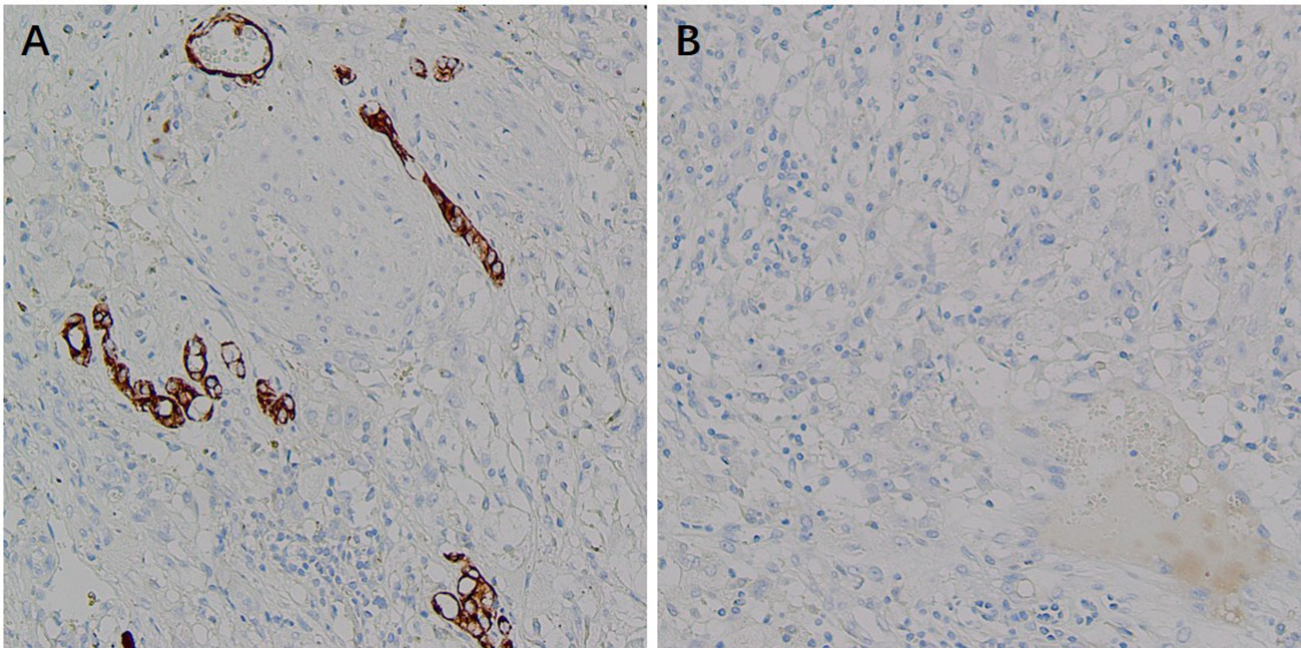
Immunohistochemical (IHC) staining of malignant renal PEComa (epithelial markers). **(A)** CK: negative staining in tumor cells. **(B)** CK7: negative staining in tumor cells.

Immunohistochemistry showed HMB45 (+) and Melan-A (+), supporting melanocytic differentiation; SMA (weak +) and Desmin (weak +) supported smooth muscle differentiation. TFE3 (+) suggested the possibility of TFE3-associated molecular alterations. PAX8 (−), RCC (−), CAIX (−), CK (−), CK7 (−), and CD117 (−) argued against renal cell carcinoma and other epithelial neoplasms. INI-1 expression was retained (+). Vimentin (+) and CD10 (+) served as supportive markers of mesenchymal origin. The Ki-67 labeling index was approximately 20%, indicating a moderate proliferative fraction. Taken together, the histomorphology and immunophenotype supported the diagnosis of malignant PEComa of the right kidney (**Figures 6**–**9**).

## 3 Discussion and conclusions

This case combines two uncommon but clinically significant entities: a malignant renal PEComa presenting with spontaneous retroperitoneal hemorrhage and Wunderlich syndrome, and a delayed duodenal perforation following retroperitoneal laparoscopic radical nephrectomy. Renal PEComas themselves are rare, and presentations with tumor rupture and massive retroperitoneal hemorrhage have only occasionally been reported (13–16). To our knowledge, reports of malignant renal PEComa complicated by duodenal injury after nephrectomy are exceedingly scarce (17), making this case of particular interest from both oncologic and surgical perspectives.

Histologically, the tumor in our patient fulfilled several high-risk criteria for malignancy, including large size, infiltrative growth with capsular rupture, necrosis, and a markedly elevated proliferative index, in line with previously proposed risk stratification schemes for PEComa (18, 19). Malignant PEComas are associated with a substantial risk of local recurrence and distant metastasis, often involving the lung, liver and bone (20, 21). These features justify aggressive local control with radical nephrectomy and careful long-term surveillance. Our case adds to the growing body of literature indicating that renal PEComas, particularly those with adverse histologic features, should be regarded as potentially aggressive neoplasms requiring prolonged oncologic follow-up.

Clinically, the initial presentation with spontaneous retroperitoneal hemorrhage and Wunderlich syndrome reflects one of the most dramatic manifestations of renal PEComa. In most series, the dominant causes of Wunderlich syndrome are renal cell carcinoma and angiomyolipoma (22–24). However, PEComa should also be considered in the differential diagnosis of hemorrhagic renal masses, especially when imaging reveals a heterogeneous lesion with intratumoral hemorrhage and no clear features of classic angiomyolipoma. In our patient, the combination of flank pain, acute anemia and a large perirenal hematoma suggested a ruptured renal neoplasm, and a retroperitoneal laparoscopic radical nephrectomy was undertaken as definitive treatment and hemostasis.

Duodenal perforation after nephrectomy is rare but associated with high morbidity and mortality, particularly when diagnosis is delayed (17). The risk is greatest in right-sided procedures, where the second and third portions of the duodenum lie in close proximity to the renal hilum, inferior vena cava and Gerota’s fascia (25). In the presence of extensive retroperitoneal hematoma, inflammation and adhesions – as in our case – anatomical planes become distorted and dissection within this “hostile” retroperitoneum may predispose to occult thermal or traction injuries to the duodenal wall (26, 27). Such injuries may not be apparent intraoperatively, even with careful inspection, and can subsequently manifest as retroperitoneal leakage, severe sepsis or generalized peritonitis.

The patient’s postoperative course in this case highlights the diagnostic challenges of identifying duodenal injury in such complex settings. Postoperatively, the patient developed sudden, severe abdominal pain with signs of peritoneal irritation. Laboratory testing suggested a persistent systemic inflammatory response, manifested by progressively worsening leukocytosis and rising procalcitonin levels. Although these findings may initially be attributed to surgical trauma or resorption of a retroperitoneal hematoma, the persistence and progression of the systemic inflammatory response should prompt concern for an occult infectious source. A key clue in this case was the marked elevation of urinary amylase, which is uncommon after simple nephrectomy and suggests leakage of pancreatic or upper gastrointestinal secretions into the retroperitoneal space. Subsequent emergent exploratory laparotomy confirmed the presence of exudative fluid in the retroperitoneum and revealed a small perforation in the descending portion of the duodenum; perforation repair, extensive drainage, and placement of a jejunal feeding tube were performed.

The duodenal perforation in this case should be clearly regarded as a postoperative complication that is difficult to completely avoid under a specific, highly complex pathological background. The fundamental driver was the primary disease—malignant renal perivascular epithelioid cell tumor with spontaneous rupture and hemorrhage—which created a profoundly inflamed retroperitoneal environment with dense adhesions. As demonstrated by preoperative imaging and intraoperative findings, capsular rupture and extensive intratumoral hemorrhage led to a large organized hematoma, resulting in tight adhesion between the renal upper pole and the descending duodenum. This severe local inflammatory response, triggered by the tumor’s biological behavior, fundamentally altered normal anatomy: tissue planes were obliterated, anatomical relationships distorted, and the duodenum became tightly incorporated into the diseased area. Radical resection performed under such “unfavorable” anatomical conditions therefore carries a substantially increased risk of duodenal injury. Importantly, this risk is better understood as the consequence of tumor-related pathological changes interacting with necessary surgical intervention, rather than an isolated “iatrogenic injury” that can be simply attributed to operative technique. Such injuries may result from unavoidable minor serosal trauma during dissection of dense adhesions, local perfusion compromise, or thermal spread from energy devices; moreover, immediate intraoperative recognition is particularly difficult given the limited field of view inherent to the retroperitoneal laparoscopic approach.

Although the risk of this complication is heightened in this specific context, it can be proactively managed and potentially reduced through prospective adjustments in surgical strategy. The experience from this case suggests that for right-sided renal upper-pole tumors associated with preoperative spontaneous bleeding, massive hematoma, or imaging features suggestive of severe adhesions, duodenal injury should be explicitly discussed as an important potential complication during preoperative planning, and thorough informed consent should be obtained. If dense inflammatory adhesions similar to those in this case are confirmed intraoperatively, early conversion to an alternative surgical approach should be considered. A transperitoneal approach (laparoscopic or open) provides a wider operative field and facilitates safe mobilization of the duodenum (e.g., via the Kocher maneuver), enabling more controlled adhesiolysis under direct visualization—potentially safer than operating within a narrow, distorted retroperitoneal space. In addition, an active intraoperative assessment “warning” mechanism should be established: after tumor resection, if there is any concern regarding duodenal integrity, surgeons should maintain a high index of suspicion and adopt a low threshold for further evaluation. Intraoperative upper gastrointestinal endoscopy (with laparoscopic assistance using insufflation and/or a methylene blue test) may be considered to exclude occult leakage, and intraoperative consultation with a general surgeon may also be warranted.

From a practical standpoint, this case emphasizes several important points for surgeons and clinicians. First, when performing retroperitoneal laparoscopic nephrectomy in the setting of a ruptured right renal tumor with large perirenal hematoma and inflammation, the duodenum should be regarded as a high-risk structure, and meticulous dissection with minimization of thermal spread is essential. Second, in the postoperative period, persistent or worsening systemic inflammation, especially in combination with markedly elevated urine amylase (hyperamylasuria), should prompt early abdominal imaging to rule out retroperitoneal gastrointestinal leakage rather than being attributed solely to “normal” postoperative changes. Third, once duodenal injury is suspected, a low threshold for surgical re-exploration is crucial, as delayed intervention is associated with significantly worse outcomes.

In summary, this case indicates that malignant renal PEComa may present with spontaneous retroperitoneal hemorrhage and Wunderlich syndrome, and its clinical manifestations can be easily confused with those of common renal tumors. During retroperitoneal laparoscopic radical nephrectomy in the setting of complex local anatomy, Surgeons should maintain a high of attention for occult duodenal injury. In addition to striving for technical precision during the procedure, a systematic risk-prevention strategy should be established, encompassing preoperative assessment, selection of the surgical approach, and intraoperative decision-making. If persistent systemic inflammatory response occurs postoperatively accompanied by a marked elevation of urinary amylase, the possibility of retroperitoneal duodenal leakage should be suspected, and imaging as well as biochemical evaluation should be performed as early as possible; surgical exploration should be undertaken in a timely manner when necessary. With prospective assessment and flexible surgical strategies, the risk of such rare but severe complications and their adverse outcomes may be reduced.

## Data Availability

The original contributions presented in the study are included in the article/supplementary material. Further inquiries can be directed to the corresponding author.

## Glossary

18F-FDG: Fluorine-18 fluorodeoxyglucose
AMACR: Alpha-methylacyl-CoA racemase
AMYL: Amylase
CAIX: Carbonic anhydrase IX
CD10: Cluster of differentiation 10
CD68: Cluster of differentiation 68
CD117: Cluster of differentiation 117 (KIT)
CK7: Cytokeratin 7
CT: Computed tomography
EMA: Epithelial membrane antigen
FDG: Fluorodeoxyglucose
H&amp;E: Hematoxylin and eosin
HMB-45: Human melanoma black 45
INI1: Integrase interactor 1 (SMARCB1)
Ki-67: Ki-67 proliferation marker
Melan-A: Melanoma antigen recognized by T cells 1 (MART-1)
MIP: Maximum-intensity projection
MyoD1: Myogenic differentiation 1
P504S: AMACR antibody (P504S)
PAX8: Paired box gene 8
PCT: Procalcitonin
PEComa: Perivascular epithelioid cell tumor
PET: Positron emission tomography
PET/CT: Positron emission tomography/computed tomography
POD: Postoperative day
RCC: Renal cell carcinoma
SIRS: Systemic inflammatory response syndrome
SMA: Smooth muscle actin
TFE3: Transcription factor E3
WBC: White blood cell count
